# Effect of Chewing Paneer and Cheese on Salivary Acidogenicity: A Comparative Study

**DOI:** 10.5005/jp-journals-10005-1128

**Published:** 2012-02-24

**Authors:** Tabassum Tayab, Kavitha Rai, Vasantha Kumari, Eapen Thomas

**Affiliations:** Senior Lecturer, Department of Pedodontics, MR Ambedkar Dental College and Hospital, Bengaluru, Karnataka, India e-mail: drtabassum2001@yahoo.co.in; Professor, Department of Pedodontics, AB Shetty Dental College Mangalore, Karnataka, India; Professor and Head, Department of Pedodontics, Sri Ramachandra Dental College and Hospital, Chennai, Tamil Nadu, India; Assistant Professor, Meenakshi Ammal Dental College, Chennai Tamil Nadu, India

**Keywords:** Cheese, Paneer, Salivary pH, Diet counseling, Protective foods

## Abstract

**Aim:** The aim was to evaluate the salivary pH reversal phenomenon by chewing paneer and processed cheese after a chocolate challenge.

**Materials and methods:** Thirty caries-free children were randomly selected and divided into 2 groups: Control group was given processed cheese (Amul) and the experimental group was given paneer (Amul) after a chocolate challenge. After determining the resting salivary pH using GC pH strips, the subjects were asked to eat the test foods and salivary pH was measured at time intervals of 5, 10, 15, 30 and 60 minutes to record the time taken for the salivary pH to return to baseline values after an acidogenic challenge.

**Results:** The data was analyzed and intergroup comparison was done using paired student’s t-test. The test meals increased salivary pH after chocolate challenge significantly from baseline values and neutralized the fall in pH after a chocolate challenge. The protective effect was evident after 5 minutes of consuming chocolate and was highest up to 30 minutes after which the salivary pH gradually fell but had not returned to baseline values even at 60 minutes.

**Conclusion:** The findings suggest that chewing of paneer like cheese abolishes the fall in salivary pH caused by sugar consumption and maybe recommended as a protective food in pediatric diet counseling.

**How to cite this article:** Tayab T, Rai K, Kumari V, Thomas E. Effect of Chewing Paneer and Cheese on Salivary Acidogenicity: A Comparative Study. Int J Clin Pediatr Dent 2012;5(1):20-24.

## INTRODUCTION

There is sufficient evidence regarding the effect of saliva in controlling plaque pH, and that stimulation of saliva by foods is an important factor in determining their acidogenic potential. This is especially important when saliva is stimulated after plaque pH is lowered by an acidogenic challenge.^1,2^ Chewing of certain foods, such as cheese promotes a rapid recovery of plaque pH following an acidogenic challenge thereby exerting a caries protective effect. (Geddes et al, 1977; Jensen et al, 1984; Rugg-Gunn et al, 1975).^3-5^

Dental caries is an infectious and nutrition-related disease. Eating patterns and especially consumption of sugary foods between meals can result in tooth decay.^6^ Diet counseling forms an important part of preventive dentistry and as dentists see more caries-prone patients they are increasingly called upon to identify and give advice on foods that inhibit the carious process, rather than, systemic nutritional counseling for developing a caries-resistant tooth. Prevention of excess sucrose consumption appears to be a reasonable component of a caries prevention program.^7^ Yet, there is presently no evidence demonstrating the effectiveness of this restrictive approach of dietary counseling on caries reduction in children due to poor compliance.

Although dairy products are proven to be caries protective foods, individuals make food choices in the context of their culture, and owing to the lack of availability and high cost of cheese in the Indian subcontinent, paneer the traditional Indian cheese was chosen as the test food in this study. Paneer is an unsweetened, unripened form of cheese made by the addition of lime juice to milk which retains the noncariogenic components of milk. It contains a higher protein and phosphate content when compared to cheese but it is not known how this affects its cariogenic potential.^8^

In the present study assessing the acidogenic potential of paneer, was designed using a standard acidogenic challenge to allow comparison of two types of cheese. The aim was to evaluate the salivary pH reversal phenomenon by chewing paneer and processed cheese after a chocolate challenge. Past research used plaque telemetry^5^ to measure acidity but we used salivary pH to develop a clinically usable patient-specific procedure for dietary counseling in children.

## MATERIALS AND METHODS

A total of 36 volunteers were randomly selected from the outpatients who reported to the Department of Pedodontics and Preventive Dentistry, Sri Ramachandra Dental College, Chennai. The inclusion criteria were healthy children ranging in age from 5 to 12 years and caries free and from whom consent was obtained. A total of 17 males and 19 females were included in the study. Subjects with caries, history to food allergies especially to dairy products and were unwilling to participate were excluded. Approval for the research was obtained from the Ethical Committee of Sri Ramachandra Dental College and University, Chennai and informed consent from each participant prior to the start of the study was obtained.

The subjects were divided into two groups: Control group was given processed cheese (Amul) and the experimental group was given paneer (Amul) after a chocolate challenge (Cadbury Dairy Milk Chocolate Bar- 12.5 gm). Since the commercially available chocolate weighs 12.5 gm the same quantity of test foods were weighed and used.

Oral prophylaxis was done for all the subjects in the study and control group 24 hours prior to study and were instructed to refrain from eating or drinking at least 2.5 hours prior to the test. They were instructed to rinse their mouth with distilled water.

## SALIVARY pH MEASUREMENTS

Unstimulated saliva was collected by Navazesh (1993) spitting method^9^ by pooling saliva for 60 seconds and then spitting in a plastic disposable container. Salivary pH was measured using GC pH strips. A single sheet of test paper was removed from the booklet. The pH strip was dipped into the saliva till it is fully wet and removed immediately. After 30 seconds the acids produced react to these pH indicators, thus leading to a colorimetric change which was compared with the color code chart and the pH value was noted whilst the paper was moist.^10^

**Fig. 1:**
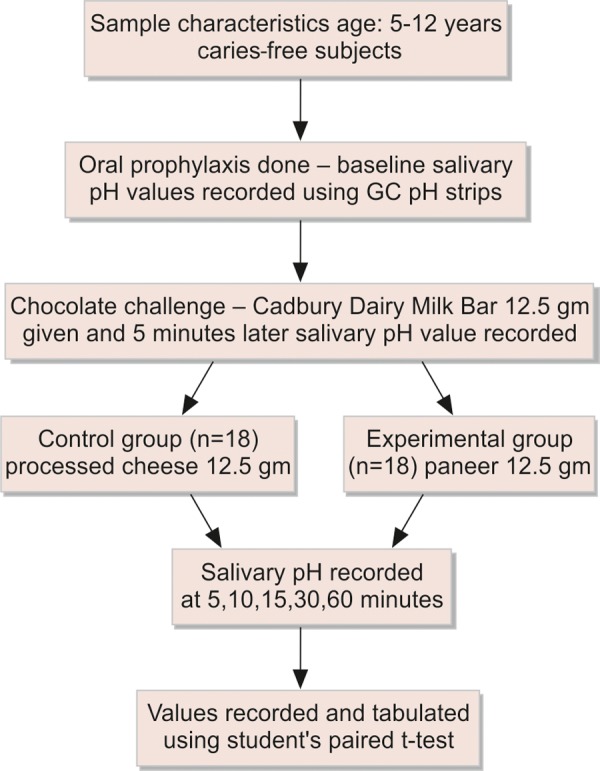
Experimental method for testing the effect of chewing paneer and processed cheese on salivary pH after chocolate challenge

After determining the resting salivary pH the subjects were asked to eat chocolate and salivary pH was measured at time intervals of 5, 10, 15, 30 and 60 minutes to record the time taken for the salivary pH to return to baseline values after an acidogenic challenge.^11,12^ The same subjects were then asked to rinse thoroughly with tap water and the baseline pH was measured as described above. After this the subjects were asked to eat chocolate again and salivary pH was measured at 5 minutes. The subjects were then instructed to eat cheese and paneer 5 minutes after the chocolate challenge and salivary pH measured at 0, 5, 10, 15, 30 and 60 minutes. All readings were taken 30 seconds after insertion of test strip in saliva (Fig. 1).

The maximum pH attained with each test food after chocolate challenge and the difference between resting salivary pH and maximum salivary pH was established. The mean salivary pH values were tabulated and significance was calculated using student’s paired t-test. The value was considered significant when p-value was less than 0.05.

### RESULTS

After eating chocolate salivary pH in both the groups showed minimum pH drop at 5 minutes and this remained significantly lower (p < 0.05) than the baseline in all of the test periods. However, the drop in salivary pH after eating chocolate when compared to the salivary pH after eating paneer was statistically significant (p < 0.001). Paneer did not make a great change in salivary pH with time. In the first minute after eating paneer salivary pH showed a significant increase (p < 0.001) from baseline. Thereafter the pH showed a small decrease after 5 minutes but stayed significantly higher than baseline value even at 60 minutes (Table 1).

There was a significant rise in salivary pH from baseline to after eating cheese (p < 0.05) for all the test periods. There was a drop in salivary pH after eating chocolate when compared to the salivary pH after eating cheese and was found to be statistically significant (p < 0.01) (Table 2).

**Graph 1:**
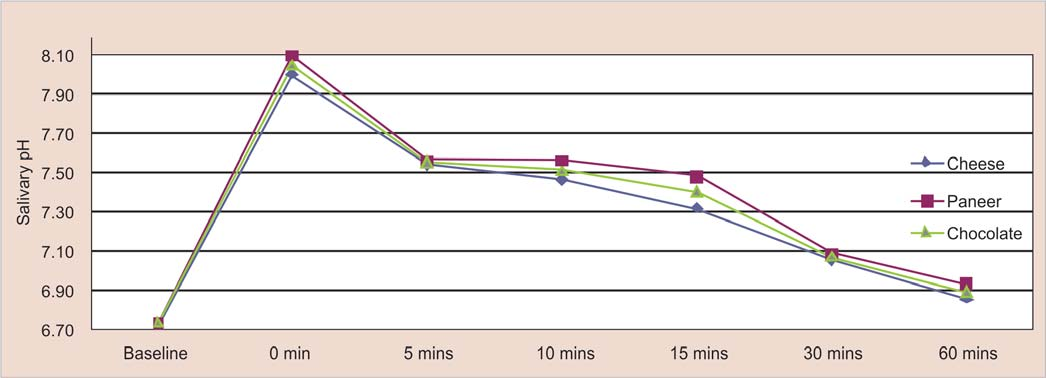
Change in mean salivary pH at different time intervals for all test foods

The mean salivary pH response to chocolate shows an initial sharp rise followed by a fall in pH reaching a minimum after 5 minutes and gradually returning to resting values after approximately 60 minutes. When paneer and cheese were chewed following chocolate a maximum rise in pH was observed but the difference was not statistically significant (p > 0.05) (Table 3). Thereafter the pH gradually fell at 5 minutes but was much higher than resting values and remained high for approximately 15 minutes following which the pH reduced but had not returned to baseline values at 60 minutes (Graph 1).

**Table 1:** **Table 1: **Salivary pH changes with paneer

*Time interval*		*Mean*		*Std dev*		*Mean difference*		*t*		*p-value*	
Baseline pH		6.92		0.28		0.178		2.637		0.017*	
pH after eating chocolate		6.74		0.24							
Baseline pH		6.92		0.28		–0.628		–5.636		<0.001*	
pH after eating paneer		7.54		0.37							
pH after eating chocolate		6.74		0.24		–0.806		–9.657		<0.001*	
pH after eating paneer		7.54		0.37							

**Table 2:** **Table 2: **Salivary pH changes with cheese

*Time interval*		*Mean*		*Std dev*		*Mean difference*		*t*		*p-value*	
Baseline pH		6.93		0.27		0.144		1.292		0.214	
pH after eating chocolate		6.79		0.44							
Baseline pH		6.93		0.27		–0.367		–2.807		0.012*	
pH after eating cheese		7.30		0.52							
pH after eating chocolate		6.79		0.44		–0.511		–4.254		0.001*	
pH after eating cheese		7.30		0.52							

**Table 3:** **Table 3: **Comparison of salivary pH values recorded in the cheese and paneer groups

*Group*		*Mean*		*Std dev*		*Mean difference*		*t*		*p-value*	
Paneer		7.54		0.37		0.244		1.620		0.115	
Cheese		7.30		0.52							

### DISCUSSION

One approach to estimate the acidogenic potential of food involves evaluation of the magnitude of the pH response following ingestion of food. Consequently, methods to measure oral pH include plaque sampling, touch electrodes and built in electrodes.^13^ A patient-specific approach using pH strips to assess the acidogenic potential of paneer and cheese was used in the present study which also aids in patient and parent diet counseling. Although salivary pH is not the only parameter that predisposes to dental caries it is an effective educational tool at the chairside and in school health education programs to educate smaller and larger groups on the nutritional and protective aspects of food as part of diet counseling.

Salivary pH is influenced by flow rate, duration of stimulation and calcium concentration, therefore, the subjects were instructed to chew all test foods for a minute to standardize the experimental conditions. Hence, we chose to record the salivary pH values after 5 minutes had elapsed since a minimum drop in pH after the chocolate challenge was seen at 5 minutes.

Prior to consumption of test foods resting salivary pH was recorded to provide baseline values against which the rise and drop in pH could be evaluated. The baseline values thus, measured were in the range of 6.4 to 7.4 and are similar to earlier reports.^14^ The results tend to confirm previous reports of salivary testing showing a subject-to-subject variation in response to test foods as individuals in a population differ considerably in salivary pH due to variation in caries susceptibility.

In the present study, a shallow drop in pH (acidic) was seen in 70% of subjects and is consistent with Stephan’s observations that sugar containing foods cause a rapid drop in plaque (oral) pH.^15^ This shallow pH response to chocolate maybe attributed to the difference in the method of assessing oral pH since, most studies assessed plaque pH. However, in 30% of children no change in pH was detected. The probable reason for this lack in change in salivary pH maybe because their resting salivary pH was higher than 7.0 thus, had better buffering capacity and was similar to Birkhed’s findings.^14^ Also the gustatory stimulus provided by eating chocolate further elevates salivary buffering capacity preventing the drop in pH and explains the rise in salivary pH seen in the first minute. High-sugar foods (chocolate, caramels) have a higher oral clearance as compared to high starch foods which maybe another contributing factor for the lack in change in salivary pH.^16,17^ In addition since the subjects had good oral hygiene with minimal plaque it may have contributed to the lack in change seen in salivary pH. It should be borne in mind that although the critical pH of 5.5 was not detected with pH strips after eating chocolate, it should not be considered as a safe food for teeth.

The rise in salivary pH due to cheese after chocolate challenge was similar to the results of Athena Papas; Rugg- Gunn et al demonstrated a pH reversal when cheese was eaten after an exposure to sugar. ^2,18^ In the present study a rise in salivary pH was seen in 82% of the subjects chewing paneer after a chocolate challenge. Harper et al have classified cheese into categories, such as fresh cream, soft, hard, etc. that reflect the differences in texture, aging, level of butterfat, casein and Ca/PO_4_ content.^19^ Evidence suggests that hard cheese demonstrated alkaline pH values as compared to soft cheese and was in contrast to our finding.^7,19-21^ Furthermore, Pickerill stated that the nature of stimulus affects composition of saliva and an acid stimulus elicited secretion of saliva of high buffering capacity with sweet being the least.^2^ This was in contrast to our findings as paneer being a bland, semi-firm and unripened form of cheese produced minimal gustatory and mechanical stimulus as compared to processed cheese. Although both processed cheese and paneer caused an alkaline pH we found that paneer aided in faster neutralization of acidic saliva and this beneficial effect lasted for a longer time although not significant. In contrast, Krobicka et al demonstrated the protective effect of cheese was still present in desalivated rats indicating the possibility of other mechanisms.^22^ Casein phosphopeptides released by the proteolysis of cheese following intake lead to the formation of casein phosphopeptide-calcium phosphate complexes (CPP-CP) which increase calcium and phosphate in plaque thereby increasing its pH.^23,24^ This maybe cited as the probable reason for recording higher pH values with paneer as it had a higher protein and Ca/PO_4_ content as compared to processed cheese. Cheese may also protect against caries by reducing the adherence of mutans streptococci to tooth surfaces.^25^ Lipids in cheese may be protective by forming a coating on enamel surfaces which can reduce demineralization of tooth enamel surfaces and/ or by an antibacterial action of fatty acids.^19^ We can extrapolate these findings to explain the alkaline pH caused by intake of paneer. ^21^

The substrate that causes a prolonged acidic pH is more detrimental to the teeth than its sugar content alone.^2^ In this study it was found that the fall in salivary pH caused by consuming chocolate reaching a maximum at 5 minutes and it took 60 minutes to revert to baseline values indicating the prolonged deleterious effect of chocolate consumption. The fall in salivary pH was rapidly reversed when followed by eating paneer and cheese and this protective effect reached a maximum at 30 minutes. However, even after an hour the salivary pH was higher than baseline values suggesting the prolonged caries protective effect of paneer and cheese.

Although the caries protective effect of cheese has been well-documented in the Western world, for the Indian subcontinent, indigenous products like paneer are more practical choices as they form a part of our traditional diet. The findings of this pilot study assessed indirectly the anti- cariogenicity of paneer thereby it maybe suggested as a final food in a meal as a caries protective measure.

Currently, most dental practitioners focus on merely providing dietary information which may be insufficient to bring about the desired change in behavior that can impact the success of nutritional counseling.^26,27^Identification of effective educational methods to help the public translate dietary recommendations into appropriate food choices is the need of the hour. Therefore, our study proposes an out of the box thinking methodology of pediatric dietary counseling with the use of pH strips as a visual educational tool to encourage acceptance by giving out positive messages of following a sugary snack with a pH reversing food like paneer. This method gives the patient a sense of being in control of his diet as he can choose foods based on pH strip values, it is easy to do and can even be done as a fun activity by children thereby arousing their interest and hence may encourage better compliance. Additionally, it can be promoted as an educational campaign in school health programs and for children in practice by formulating quotes like ‘Chew your way to pearly whites with paneer’ or ‘Chew your way to oral health naturally... chew paneer’.

## CONCLUSION

In this study paneer like processed cheese reversed the drop in salivary pH levels after a chocolate challenge and the salivary pH levels were elevated for 60 minutes indicating that the protective effect of paneer lasted for over an hour. Hence, paneer can be recommended as a final food in a meal as a caries protective measure. Since, paneer is homemade and is a part of Indian diet it is a more economical choice than cheese for the Indian subcontinent. Hence, the findings of this study have demonstrated the method of creating a favorable environment to individual choice of healthier diets.

### Recommendations to Parents

Allow children to choose foods by conducting the pH strip test. Dietary choices are largely governed by taste and it is impractical to suggest a sugar-free diet to a young child. Instead all healthcare providers should take cognizance of this new trend of chewing paneer or cheese after a sugary snack.

### To Dental Health Professionals

To routinely use pH strips during diet counseling, so that didactic information is supplanted with a patient-specific visual tool. This method enhances patient’s comprehension of the effect of his food choices on his teeth and how to negate its deleterious effects with protective foods.
